# Roles and Cellular Localization of GBP2 and NAB2 During the Blood Stage of Malaria Parasites

**DOI:** 10.3389/fcimb.2021.737457

**Published:** 2021-09-15

**Authors:** Mamoru Niikura, Toshiyuki Fukutomi, Jiro Mitobe, Fumie Kobayashi

**Affiliations:** ^1^Department of Infectious Diseases, Kyorin University School of Medicine, Tokyo, Japan; ^2^Department of Pharmacology and Toxicology, Kyorin University School of Medicine, Tokyo, Japan; ^3^Department of Environmental Science, School of Life and Environmental Science, Azabu University, Kanagawa, Japan

**Keywords:** malaria, RNA-binding protein, NAB2, GBP2, quality control, export

## Abstract

The quality control and export of mRNA by RNA-binding proteins are necessary for the survival of malaria parasites, which have complex life cycles. Nuclear poly(A) binding protein 2 (NAB2), THO complex subunit 4 (THO4), nucleolar protein 3 (NPL3), G-strand binding protein 2 (GBP2) and serine/arginine-rich splicing factor 1 (SR1) are involved in nuclear mRNA export in malaria parasites. However, their roles in asexual and sexual development, and in cellular localization, are not fully understood. In this study using the rodent malaria parasite, *Plasmodium berghei*, we found that NAB2 and SR1, but not THO4, NPL3 or GBP2, played essential roles in the asexual development of malaria parasites. By contrast, GBP2 but not NPL3 was involved in male and female gametocyte production. THO4 was involved in female gametocyte production, but had a lower impact than GBP2. In this study, we focused on GBP2 and NAB2, which play important roles in the sexual and asexual development of malaria parasites, respectively, and examined their cellular localization. GBP2 localized to both the nucleus and cytoplasm of malaria parasites. Using immunoprecipitation coupled to mass spectrometry (IP-MS), GBP2 interacted with the proteins ALBA4, DOZI, and CITH, which play roles in translational repression. IP-MS also revealed that phosphorylated adapter RNA export protein (PHAX) domain-containing protein, an adaptor protein for exportin-1, also interacted with GBP2, implying that mRNA export occurs *via* the PHAX domain-containing protein pathway in malaria parasites. Live-cell fluorescence imaging revealed that NAB2 localized at the nuclear periphery. Moreover, IP-MS indicated that NAB2 interacted with transportin. RNA immunoprecipitation coupled to RNA sequencing revealed that NAB2 bound directly to 143 mRNAs, including those encoding 40S and 60S ribosomal proteins. Our findings imply that malaria parasites use an evolutionarily ancient mechanism conserved throughout eukaryotic evolution.

## Introduction

Malaria parasites are unicellular eukaryotes that belong to the genus *Plasmodium* in the phylum Apicomplexa. Malaria parasites have complex life cycles, alternating between female Anopheles mosquitoes and vertebrate hosts. The quality control and export of mRNA, as well as the post-transcriptional regulation of gene expression by RNA-binding proteins, play important roles in the life cycles of malaria parasites (Mair et al., 2006; Guerreiro et al., 2014).

In Opisthokonta, such as yeast, the mRNA export receptor Mex67/Mtr2 interacts with nuclear pore complex proteins and plays a pivotal role during the terminal step of nuclear mRNA export (Katahira, 2015; Wickramasinghe and Laskey, 2015; Xie and Ren, 2019). The mRNA export receptor is recruited by adaptor proteins such as nuclear poly(A) binding protein 2 (NAB2), yeast RNA annealing protein (YRA1) and three serine/arginine-rich (SR) proteins, including nucleolar protein 3 (NPL3), G-strand binding protein 2 (GBP2) and hypothetical RNA-binding protein (HRB1) (Hackmann et al., 2014; Heath et al., 2016; Zander and Krebber, 2017). Apicomplexans do not belong to the phylogenetic group Opisthokonta. Orthologs of Mex67 and Mtr2 are absent from the genomes of malaria parasites (Serpeloni et al., 2011; Avila et al., 2018), in which only five or six nucleoporins (Nups) have been identified (Kehrer et al., 2018). Despite this, malaria parasites possess genes encoding all of the adaptor proteins required for Mex67/Mtr2 recruitment (Tuteja and Mehta, 2010).

NAB2 and YRA1 play essential roles in mRNA export in *Saccharomyces cerevisiae*. YRA1 enhances the interaction between NAB2 and Mex67. NAB2 contains an N-terminal PWI domain and a C-terminal CCCH-type zinc finger motif, similar to yeast NAB2 (Tuteja and Mehta, 2010). Moreover, a nuclear localization signal (NLS), RX_2–5_PY NLS (PY-NLS) (Soniat et al., 2013), is present in NAB2 according to the PlasmoDB database (www.plasmodb.org/). THO complex subunit 4 (THO4) is a homologue of yeast YRA1 in malaria parasites. THO4 is characterized by its central RNA-binding domain (Tuteja and Mehta, 2010). However, the functions of NAB2 and THO4 in *Plasmodium* species remain unknown.

Homologs of NAB2, GBP2, SR1, and PHAX have been identified in yeast and humans (Tuteja and Mehta, 2010). In *S. cerevisiae*, individual SR proteins such as NPL3, GBP2, and HRB1 are not essential (Zander and Krebber, 2017). NPL3, GBP2 and SR1 contain conserved RNA recognition motifs. SR1 is essential for asexual development in *Plasmodium falciparum* (Eshar et al., 2012). On the other hand, in the rodent malaria parasite *Plasmodium berghei* ANKA, GBP2 is primarily involved in sexual development (Niikura et al., 2020). However, the roles of NPL3, GBP2, and SR1 in malaria parasites are not fully understood.

In this study, we investigated the roles of NAB2, THO4, NPL3, GBP2, and SR1 during asexual and sexual development in *P. berghei* ANKA *via* reverse genetics. We found that NAB2 and SR1, but not THO4, NPL3 or GBP2, played essential roles in the asexual development of malaria parasites. On the other hand, THO4 and GBP2, but not NPL3, were involved in gametocyte production. In particular, GBP2 played a pivotal role in sexual development. To investigate the roles of GBP2 and NAB2 during the sexual and asexual developmental stages, respectively, we examined their cellular localization using *P. berghei* ANKA expressing GBP2 or NAB2 fused to the fluorescent protein mCherry. Moreover, we identified interacting proteins using immunoprecipitation coupled to mass spectrometry (IP-MS) and binding mRNAs using RNA immunoprecipitation coupled to RNA sequencing (RIP-seq).

## Materials and Methods

### Mouse Studies and Ethics

Five- to six-week-old female C57BL/6J (B6) mice were purchased from CLEA Japan Inc. (Tokyo, Japan). The experiments were approved (#221) by the Experimental Animal Ethics Committee of Kyorin University School of Medicine (Tokyo, Japan), and all experimental animals were kept at the animal facility in a specific-pathogen-free unit with sterile bedding, food, and water.

The infection studies included frequent observations to determine humane endpoints, at which mice were unable to ambulate sufficiently to obtain water or food. At the indicated time points, mice were euthanized by cervical dislocation under isoflurane or pentobarbital sodium anesthesia (N = 64). All experiments were designed to minimize suffering. When illness or death was expected due to experimental infections, mice were visually checked by investigators at least twice daily (including weekends and holidays). Mice that exhibited signs of neurological distress, such as cerebral paralysis or depression, were humanely sacrificed by cervical dislocation under isoflurane anesthesia and scored as deaths (N = 24). No mice died before meeting the criteria for euthanasia. The investigators who conducted the experiments had completed the Experimental Animal Ethics Committee training course on animal care and handling.

### Parasites and Infection

The *p230*-deleted *P. berghei* was generated by the previous study (Niikura et al., 2017) and used as control parasites. *p230* locus (PBANKA_030600) is not an essential gene in the complete life cycle of *P. berghei* (Janse et al., 2006). The *gbp2* (PBANKA_120500)-deleted *P. berghei* (Δ*gbp2* parasites) was generated by the previous study (Niikura et al., 2020). Malaria parasites were stored as frozen stocks in liquid nitrogen. Infected erythrocytes of transfected parasites were generated in donor mice inoculated intraperitoneally with frozen stocks of parasites. The donor mice were monitored for parasitemia daily and bled for experimental infection during periods in which the level of parasitemia increased. Experimental mice were infected intravenously with 1 × 10^4^ infected erythrocytes or 5 × 10^6^ to 5 × 10^7^ purified mature schizonts harvested by Nycodenz density gradient centrifugation of a given parasite strain.

### Transfection

To generated *nab2*-, *tho4*-, *npl3*- and *sr1*-deleted *P. berghei* ANKA, the gene-targeting vectors for *nab2* (PBANKA_1122000), *tho4* (PBANKA_1230500), *npl3* (PBANKA_0506600) and *sr1* (PBANKA_1232100) were prepared by PCR (Ecker et al., 2006; Niikura et al., 2020). Briefly, the 5’ and 3’ flanking regions of the open reading frame (ORF) of target genes were amplified by PCR. The PCR products were annealed to either side of the *human dihydrofolate reductase* (*hdhfr*)-expressing cassette and amplified by PCR using gene-specific primers (**Supplementary Table S1**, **Figure S1**). The gene-targeting vectors were introduced into the ORFs of target genes by double-crossover homologous recombination (**Supplementary Figure S1**). To generate transgenic parasites expressing mCherry-fused GBP2 or mCherry-fused NAB2, the gene-targeting vectors for *gbp2* (PBANKA_120500) or *nab2* (PBANKA_1122000) were prepared by PCR. The PCR products were annealed to either side of the red fluorescent protein gene (*mCherry*)-*hdhfr*-expressing cassette and amplified by PCR using gene-specific primers (**Supplementary Table S1**, **Figures S2A, B**). The gene-targeting vectors were introduced into the 3’ flanking regions of ORFs of target genes by double-crossover homologous recombination (**Supplementary Figures S2A, B**). To generate transgenic parasites expressing mCherry-fused NAB2 and GFP-fused NUP205, the gene-targeting vectors for *nup205* (PBANKA_1140100) were prepared by PCR. The PCR products were annealed to either side of the green fluorescent protein gene (*gfp*)-mutated *human deoxyhypusine synthase* (*hdhps*)-expressing cassette (Kaneko et al., 2015) and amplified by PCR using gene-specific primers (**Supplementary Table S1**, **Figures S2C**). The gene-targeting vectors were introduced into the 3’ flanking regions of ORFs of target genes by double-crossover homologous recombination (**Supplementary Figures S2C**). Transfection was performed using an Amaxa Basic Parasite Nucleofector Kit (Amaxa GmbH, Cologne, Germany) according to the manufacturer’s protocol (Niikura et al., 2013; Niikura et al., 2018).

### Genomic PCR

To generate gene-targeting vectors and confirm the introduction of gene-targeting vectors into target genes, genomic PCR was performed as described previously (Niikura et al., 2013). Thirty-five cycles of PCR were performed on a C1000 thermal cycler (Bio-Rad, Hercules, CA, USA). Each cycle consisted of denaturation at 98°C for 15 s, annealing at 55°C for 15 s, and extension at 68°C for 1–6 min. The PCR products were then analyzed on a 1% (w/v) agarose gel and stained with ethidium bromide.

### Parasitemia

Methanol-fixed tail-blood smears, stained with 3% Giemsa and diluted with phosphate buffer (pH 7.2) for 45 min, were subjected to microscopic examination. The number of infected erythrocytes (out of 250 erythrocytes) was enumerated when the level of parasitemia and gametocytemia exceeded 10%, while 1 × 10^4^ erythrocytes were examined in mice with lower levels of parasitemia and gametocytemia. The parasitemia and gametocytemia percentage values were calculated as follows: [(number of infected erythrocytes) ÷ (total number of erythrocytes)] × 100.

### Evaluation of Gametocyte Production *In Vitro*

To evaluate gametocyte production, early trophozoite stage malaria parasites were obtained from B6 mice exhibiting 1–2% parasitemia. Infected erythrocytes were incubated for 28 h in a 12-well plate. Methanol-fixed blood smears, stained with 3% Giemsa diluted in phosphate buffer (pH 7.2) for 45 min, were subjected to microscopic examination. Erythrocytes infected with mature schizonts containing 4–15 merozoites, and mature gametocytes showing sex-specific features such as nuclear enlargement, were counted as described previously (Niikura et al., 2020). The distribution of pigment granules throughout the cytoplasm, and enlargement of cells, were also assessed (Niikura et al., 2020). The proportions of male and female gametocytes were determined in at least 300 infected erythrocytes. The proportions of male and female gametocytes were calculated as follows: [(number of male or female gametocytes) ÷ (total number of schizonts plus male and female gametocytes)] × 100.

### Fluorescence Live Cell Imaging

Nuclear DNA was stained using Hoechst 33342 dye (Invitrogen, Waltham, MA). To examine the localization of NAB2::mCherry, MitoBright LT Green (Dojindo Laboratories, Kumamoto, Japan) was added to culture medium at 100 nM and incubated for 15 min at 37°C. Next, Hoechst 33342 was added to the culture at a concentration of 1 µg/mL. The staining medium was removed after the incubation, and fresh medium was added. Brightfield and fluorescence micrographs were captured at 1000× magnification using an All-in-One Fluorescence Microscope (BZ-X800; KEYENCE Japan, Osaka, Japan).

### Protein IP

Infected erythrocytes were transferred to RPMI1640 medium supplemented with 25% fetal bovine serum, 0.05 mg/mL penicillin and 0.05 mg/mL streptomycin. The infected erythrocytes were incubated for 22 h in 90% N_2_, 5% CO_2_ and 5% O_2_. Mature schizonts and gametocytes were harvested by Nycodenz density gradient centrifugation, as described previously (Niikura et al., 2020). Proteins were extracted using Mammalian Protein Extraction Reagent (Thermo Fisher Scientific, Waltham, MA) according to the manufacturer’s protocol. Protein IP in transgenic parasites expressing mCherry fused to GBP2 or NAB2 was performed using GFP- or RFP-Trap Agarose and a GFP-Trap-A kit, according to the manufacturer’s instructions (Chromotek, Planegg, Germany).

### MS

MS was performed as described previously (Niikura et al., 2018; Niikura et al., 2020). The database search engines Proteome Discoverer 1.4 (Thermo Scientific) and MASCOT 2.6 (Matrix Science) were used to identify and quantify proteins from the MS, MS/MS and reporter ion spectra of the peptides. Peptide mass data were matched by searching the protein database (PlasmoDB-46_PbergheiANKA.fasta), downloaded from PlasmoDB (updated November 4, 2019). The false discovery rate (FDR) (Wang et al., 2009) was calculated by peptide sequence analysis using Percolator software (Käll et al., 2007). High-confidence peptide identifications were obtained by setting a target false discovery rate threshold of ≤ 1.0% at the peptide level. The mass spectrometry proteomics data have been deposited in the ProteomeXchange Consortium *via* the PRIDE (Perez-Riverol et al., 2019) partner repository with the dataset identifier PXD027302. Proteins exhibiting at least three peptide spectral matches were excluded.

### RIP-Seq

Following protein IP, RNA was isolated using an RIP-Assay Kit (Medical & Biological Laboratories, Tokyo Japan) according to the manufacturer’s instructions. RNA from two independent RNA IP assays was prepared to generate cDNA using a SMART-Seq V4 Ultra Low Input RNA Kit for sequencing (Takara, Shiga, Japan). cDNA libraries prepared using Nextera DNA Flex Library Prep Kits (Illumina K.K., Tokyo, Japan) were analyzed using an Illumina NextSeq500 (Illumina K.K.) at FASMAC (Kanagawa, Japan). Data were matched by searching the database (PlasmoDB-47_PbergheiANKA.fasta) downloaded from PlasmoDB (updated April 23, 2020).

### Statistical Analysis

For time-series comparisons, one- and two-way ANOVAs with Fisher’s protected least significant difference (PLSD) *post hoc* test were performed using Statcel program (OMS, Saitama, Japan). *P*-values < 0.05 were considered statistically significant.

## Results

### NAB2 and SR1, but Not THO4, NPL3 or GBP2, Were Essential for Survival During Asexual Development in Malaria Parasites

**Figure 1:**
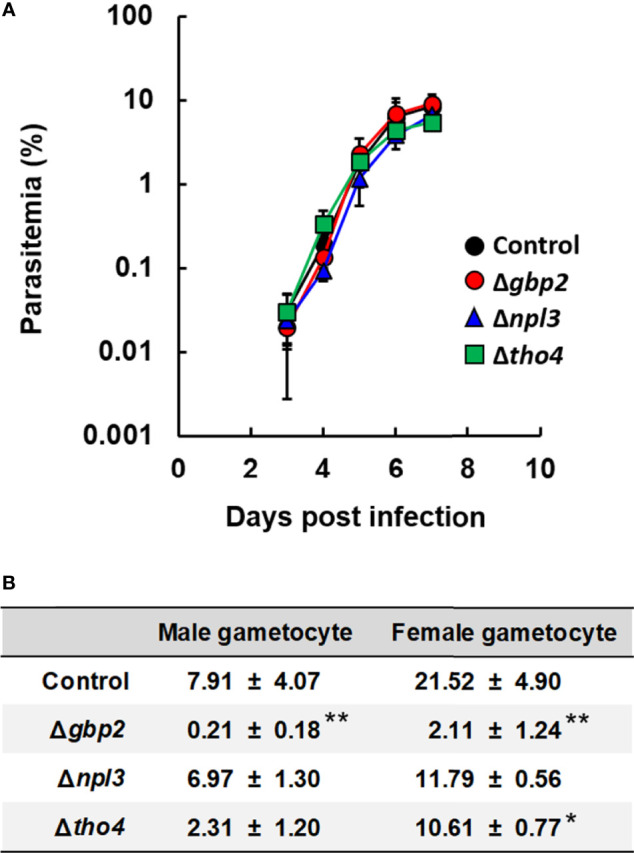
Effect of RNA-binding protein deletion on the asexual and sexual development of *P. berghei* ANKA. **(A)** Time course of parasitemia. Female C57BL/6 (B6) mice were infected with 1 × 10^4^ erythrocytes inoculated with *P. berghei* ANKA with *gbp2*, *npl3*, or *tho4* deletion (Δ*gbp2*, Δ*npl3*, or Δ*tho4* parasites, respectively). As a control, *P. berghei* ANKA with a *p230* deletion was inoculated intravenously into mice. Results are expressed as means ± standard deviation (SD) of three mice. Experiments using three mice were performed in duplicate. **(B)** Percentages of mature male and female gametocytes. Erythrocytes infected with malaria parasites were incubated for 28h. The percentages of male and female gametocytes were calculated as follows: [(number of male or female gametocytes) ÷ (total number of schizonts plus male and female gametocytes)] × 100. Results are expressed as the mean ± standard deviation of three independent experiments. * indicates a significant difference compared with the control parasites (Tukey-Kramer and Dunnett tests). ** indicates a significant difference compared with the control, Δ*npl3*, and Δ*tho4* parasites (Tukey-Kramer and Dunnett tests).

We first investigated the effects of *nab2*, *tho4*, *npl3*, *gbp2* and *sr1* deletion on the asexual development of malaria parasites. In our previous study, we generated *P. berghei* ANKA *gbp2* deletion mutants (Δ*gbp2*) (Niikura et al., 2020). In this study, we attempted to generate *P. berghei* ANKA *nab2*, *tho4*, *npl3*, and *sr1* deletion mutants (Δ*nab2*, Δ*tho4*, Δ*npl3*, and Δ*sr1*, respectively) by introducing gene-targeting vectors into the *P. berghei* ANKA genome (**Supplementary Figure S1**). In *P. berghei* knockout (PlasmoGEM) growth phenotypes, data on these mutants are not shown (PlasmoDB). By contrast, the transposon screen in *P. falciparum* showed THO4, NPL3, and SR1 to be dispensable during the asexual development of malaria parasites (PlasmoDB). In the transposon screen of *P. falciparum*, data for NAB2 and GBP2 were between dispensable and essential for asexual development (PlasmoDB). Δ*nab2* and Δ*sr1* mutants could not be generated, implying that NAB2 and SR1 are essential for the asexual development of malaria parasites. However, Δ*tho4* and Δ*npl3* mutants were successfully generated (**Supplementary Figures S1B, C**). Δ*tho4* and Δ*npl3* were inoculated intravenously into mice; their growth was monitored *in vivo* and compared with that of the control and Δ*gbp2*. The courses of parasitemia in mice infected with Δ*tho4* or Δ*npl3* were comparable with those in mice infected with the control or Δ*gbp2* parasites (**Figure 1A**). These results imply that *tho4*, *npl3*, and *gbp2* deletions did not affect the asexual development of the malaria parasites.

### Male and Female Gametocyte Production Was Decreased in *gbp2* Deletion Mutants

Previously, we found that *gbp2* deletion affects sexual development in *P. berghei* ANKA (Niikura et al., 2020). Therefore, we investigated the effects of *tho4* and *npl3* deletion on gametocyte production in malaria parasites. The percentage of male gametocytes in cultured Δ*tho4* and Δ*npl3* parasites was comparable to the percentage in control parasites (**Figure 1B**). On the other hand, the percentage of female gametocytes was lower in cultured Δ*tho4* parasites than in control parasites (**Figure 1B**). However, the percentages of both male and female gametocytes were higher in cultured Δ*tho4* and Δ*npl3* parasites than in Δ*gbp2* parasites (**Figure 1B**). These findings imply that GBP2 is involved in male and female gametocyte production in malaria parasites.

### Cellular Localization of GBP2 and NAB2 in Malaria Parasites

Our results imply that NAB2 and SR1 are essential for the asexual development of malaria parasites. SR1, but not NAB2, is localized to the nucleus and bound to RNAs in malaria parasites (Eshar et al., 2015). We showed that GBP2 plays a more important role than THO4 and NPL3 in male and female gametocyte production. However, the cellular localization of GBP2 and GBP2-binding RNAs is unknown. Therefore, to elucidate the quality control and export of mRNA by RNA-binding proteins, we focused on GBP2 and NAB2, which play important roles in the sexual and asexual development of malaria parasites, respectively. To investigate the cellular localization of GBP2 and NAB2, we generated transgenic parasites expressing GBP2 or NAB2 fusion proteins (GBP2::mCherry or NAB2::mCherry, respectively) (**Supplementary Figures S2A, B**). The mCherry tag was introduced at the C-terminus of endogenous GBP2 or NAB2. *gbp2::mCherry* and *nab2::mCherry* expression was controlled by the endogenous *gbp2* and *nab2* native promoters, respectively. Both the GBP2::mCherry and NAB2::mCherry mutant lines were successfully generated (**Figures S2A, B**) and expressed the fusion protein (**Tables 1** and **2**).

**Table 1:** Results of immunoprecipitation coupled to mass spectrometry in GBP2::mCherry parasites.

Accession	Description	ΣCoverage	Σ# Proteins	Σ# Unique Peptides	Σ# Peptides	Σ# PSMs
PBANKA_1360300	DNA/RNA-binding protein Alba 4, putative	37.17	1	12	12	67
PBANKA_1205000	single-strand telomeric DNA-binding protein GBP2, putative	30.04	1	7	7	65
PBANKA_0408400	phosphoglycerate mutase, putative	14.50	1	15	15	31
PBANKA_0501000	reticulocyte binding protein, putative	6.69	8	11	17	29
PBANKA_1359200	DNA/RNA-binding protein Alba 2, putative	29.21	1	6	6	26
PBANKA_1439200	polyadenylate-binding protein 1, putative	17.39	1	13	13	24
PBANKA_1202700	RNA-binding protein, putative	15.24	1	9	9	19
PBANKA_0817700	RNA-binding protein musashi, putative	22.09	1	7	7	16
PBANKA_0704700	conserved Plasmodium protein, unknown function	17.86	1	7	7	15
PBANKA_1217700	ATP-dependent RNA helicase DDX6	22.63	1	9	9	15
PBANKA_0600351	reticulocyte binding protein, putative	2.45	3	1	7	14
PBANKA_0506100	PHAX domain-containing protein, putative	5.35	1	8	8	14
PBANKA_1234500	FoP domain-containing protein, putative	28.27	1	5	5	12
PBANKA_1301300	trailer hitch homolog	24.63	1	7	7	12
PBANKA_1214700	conserved Plasmodium protein, unknown function	11.34	1	7	7	9

Proteins were extracted from GBP2::mCherry schizont- and gametocyte-enriched cultures after culturing for 22 h. Proteins with at least three peptide spectral matches and a fold change ≥ 2.5 compared with the controls are listed. Control experiments comprising immunoprecipitation in wild-type P. berghei ANKA using anti-mCherry beads coupled to mass spectrometry and GBP2::mCherry using anti-GFP beads coupled to mass spectrometry. Experiments were performed in triplicate. The displayed results are the sum of three independent experiments.

**Table 2:** Results of immunoprecipitation coupled to mass spectrometry in NAB2::mCherry parasites.

Accession	Description	ΣCoverage	Σ# Proteins	Σ# Unique Peptides	Σ# Peptides	Σ# PSMs
PBANKA_1122000	nuclear polyadenylated RNA-binding protein NAB2, putative	51.24	2	26	26	149
PBANKA_0306800	ATP-dependent RNA helicase UAP56, putative	41.20	1	15	15	50
PBANKA_1126400	transportin, putative	16.47	2	16	16	43
PBANKA_0830000	RNA-binding protein, putative	54.48	1	11	11	41
PBANKA_1439200	polyadenylate-binding protein 1, putative	21.46	1	16	16	41
PBANKA_0817700	RNA-binding protein musashi, putative	29.94	1	10	10	35
PBANKA_0824800	polyadenylate-binding protein 2, putative	39.70	1	7	7	31
PBANKA_1234500	FoP domain-containing protein, putative	38.22	1	7	7	29
PBANKA_1444100	T-complex protein 1 subunit gamma, putative	26.01	1	13	13	23
PBANKA_0621400	RNA-binding protein, putative	3.80	1	6	6	16
PBANKA_1242500	ATP-dependent RNA helicase DDX41, putative	14.56	1	10	10	16
PBANKA_0307800	conserved Plasmodium protein, unknown function	19.15	1	5	5	14
PBANKA_0917200	RNA-binding protein s1, putative	11.11	1	2	2	14
PBANKA_0402100	spindle pole body protein, putative	0.66	1	1	1	14
PBANKA_0112200	myosin E, putative	11.95	1	8	8	14
PBANKA_0523100	eukaryotic initiation factor 4A-III, putative	16.92	1	5	5	13
PBANKA_0919100	parasitophorous vacuolar protein 1	12.63	1	5	5	12
PBANKA_1462700	THO complex subunit 2, putative	2.69	1	6	6	11
PBANKA_1425000	RNA-binding protein, putative	3.20	1	3	3	11
PBANKA_0917900	26S protease regulatory subunit 6A, putative	19.07	1	6	6	11
PBANKA_0621600	conserved Plasmodium protein, unknown function	7.76	1	5	5	10
PBANKA_1035200	LCCL domain-containing protein	0.77	1	1	1	10
PBANKA_1024000	thioredoxin-like protein, putative	4.14	1	3	3	10
PBANKA_1241700	transmembrane emp24 domain-containing protein, putative	5.24	1	1	1	10
PBANKA_1359300	VPS13 domain-containing protein, putative	0.96	1	5	5	10
PBANKA_0101900	RNA-binding protein, putative	8.16	1	4	4	9
PBANKA_1454600	conserved Plasmodium protein, unknown function	3.45	1	2	2	9
PBANKA_1034000	conserved Plasmodium protein, unknown function	6.49	1	1	1	9
PBANKA_0938600	casein kinase 2, alpha subunit	19.70	1	5	5	9
PBANKA_1034900	pre-mRNA-splicing factor 38B, putative	1.83	1	1	1	9

Proteins were extracted from NAB2::mCherry schizont- and gametocyte-enriched cultures after incubation for 22 h. Proteins with at least three peptide spectral matches and a fold change ≥ 2.5 compared with the controls are listed. Control experiments comprising immunoprecipitation of wild-type P. berghei ANKA using anti-mCherry beads coupled to mass spectrometry and of NAB2::mCherry using anti-GFP beads coupled to mass spectrometry. Experiments were performed in triplicate. The results are the sums of three independent experiments.

**Figure 2:**
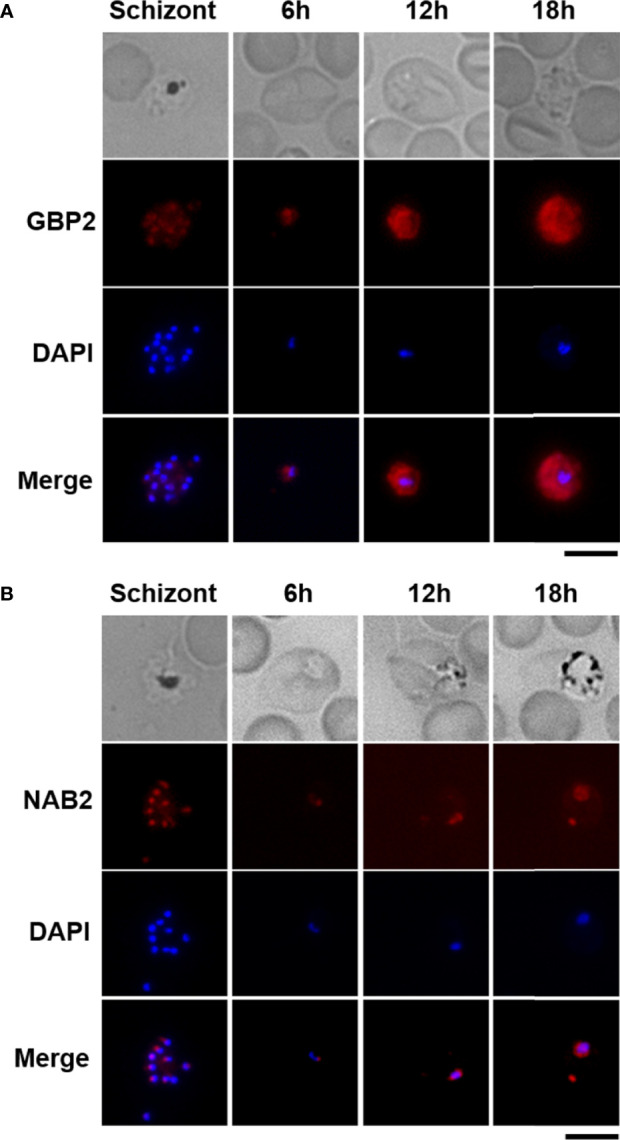
Live-cell fluorescence imaging of GBP2::mCherry- and NAB2::mCherry-expressing parasites. Erythrocytes infected with *P. berghei* ANKA schizonts at 22 h after incubation were analyzed (Schizont). Female B6 mice were infected intravenously with 5 × 10^6^ to 5 × 10^7^ mature schizonts harvested by Nycodenz density gradient centrifugation of a given parasite strain. Erythrocytes infected with malaria parasites at 6 h (ring form), 12 h (trophozoite), and 18 h (late trophozoite) after inoculation were analyzed (6, 12, and 18 h). At least 50 infected erythrocytes were analyzed, and the same fluorescence pattern was observed in all infected erythrocytes. **(A)** Transgenic parasites expressing mCherry-fused GBP2. **(B)** Transgenic parasites expressing NAB2 fused to mCherry. Scale bar = 5 µm. Experiments were performed in triplicate. Representative data are shown.

To examine the cellular localization of GBP2 and NAB2, we performed live-cell fluorescence imaging of cultured GBP2::mCherry and NAB2::mCherry schizonts and infected erythrocytes obtained from mice at 6 h (ring form), 12 h (trophozoite) and 18 h (late trophozoite) post-inoculation with GBP2::mCherry and NAB2::mCherry schizonts (**Figure 2**). The mCherry signal was distributed throughout GBP2::mCherry parasite cells at all development stages (**Figure 2A**). In NAB2::mCherry parasites, a single fluorescent spot representing the mCherry signal was present at the nuclear periphery in cultured schizonts and schizont-infected erythrocytes collected at 6 h (ring form) post-inoculation (**Figure 2B**). At 12 h (trophozoite) and 18 h (late trophozoite) post-inoculation, speckled mCherry signals were present at the nuclear periphery in NAB2::mCherry cells (**Figure 2B**). A spot of mCherry fluorescence was also detected in the cytoplasm of NAB2::mCherry parasites at 18 h (late trophozoite) post-inoculation (**Figure 2B**). These findings imply that the cellular locations of GBP2 and NAB2 in malaria parasites differ.

### NAB2 Localizes Mainly to the Inside of the Nuclear Membrane in Malaria Parasites

**Figure 3:**
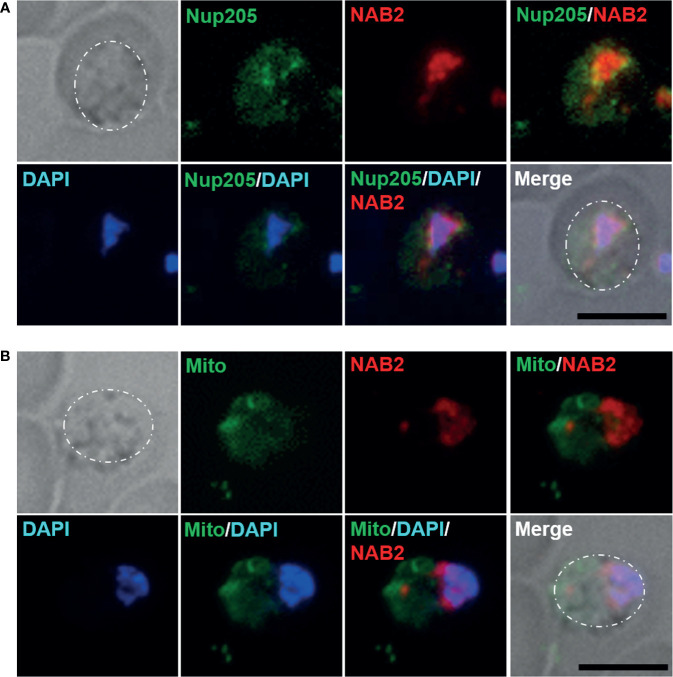
Cellular localization of NAB2 in *P. berghei* ANKA. Female B6 mice were infected with 5 × 10^6^ to 5 × 10^7^ schizonts of transgenic *P. berghei* ANKA expressing the NAB2–mCherry fusion protein (NAB2::mCherry) or NAB2::mCherry parasites expressing NUP205 fused to GFP. Erythrocytes infected with malaria parasites at 18 h (late trophozoite) after inoculation were analyzed. At least 50 infected erythrocytes were analyzed, and the same fluorescence pattern was observed in all infected erythrocytes. **(A)** NAB2::mCherry parasites expressing NUP205 fused to GFP. *P. berghei* ANKA during the late trophozoite stage are shown. **(B)** MitoBright LT Green-stained NAB2::mCherry parasites. *P. berghei* ANKA during the late trophozoite stage are shown. Scale bar = 5 µm. Dotted lines indicate malaria parasites. Experiments were performed in triplicate. Representative data are shown.

Nuclear pore complexes are composed of Nups, of which five or six have been identified in *Plasmodium* species to date (Kehrer et al., 2018). In *P. berghei*, Nup138, Nup205, Nup221, Nup313, and Nup637 have been identified as potential Nups with frequent phenylalanine–glycine repeats that localize to the nuclear periphery (Xie and Ren, 2019). To determine whether NAB2 localizes to the inside of the nuclear membrane in malaria parasites, we generated a NAB2::mCherry strain expressing NUP205 fused to green fluorescent protein (GFP) (**Supplementary Figure S2C**). Because NUP205 fluorescence increases with the size of the nucleus in *P. berghei* (Xie and Ren, 2019), we analyzed parasites during the late trophozoite stage (18 h post-inoculation). Live-cell fluorescence imaging revealed that the GFP signal was localized mainly to the nuclear periphery in *P. berghei* ANKA trophozoites (**Figure 3A**), implying that NAB2 localized to the inner nuclear membrane in *P. berghei* ANKA. To investigate whether the fluorescent signal of mCherry detected in the cytoplasm represented localization to mitochondria, NAB2::mCherry parasites were stained with mitotracker; the mCherry signal did not overlap that of mitochondria (**Figure 3B**).

### Identification of GBP2- and NAB2-Interacting Proteins in Malaria Parasites

To investigate which proteins interact with GBP2 and NAB2, we performed protein IP using anti-mCherry beads and identified the proteins bound to GBP2 and NAB2 by MS. Proteins were extracted from mature schizonts and gametocytes harvested by Nycodenz density gradient centrifugation. IP-MS using anti-mCherry beads in wild-type *P. berghei* ANKA, as well as anti-GFP beads in the GBP2::mCherry and NAB2::mCherry strains, were performed to provide controls.

In three independent comparative proteomics analyses of GBP2, 175, 191, and 236 proteins were detected. Among them, 15 proteins with at least three peptide spectral matches and a fold change ≥ 2.5 compared with the controls among three independent experiments were analyzed further (**Table 1**). Nuclear and cytoplasmic proteins—such as FoP domain-containing protein (PBANKA_1234500), which is a homolog of Friend of Prmt1 (van Dijk et al., 2010)—and polyadenylate-binding protein 1 (PBANKA_1439200) (Minns et al., 2018), respectively, were detected by IP-MS of GBP2::mCherry (**Table 1**). Moreover, the gametocyte-related proteins DNA/RNA-binding protein Alba (ALBA) 2 and 4 (PBANKA_1359200 and PBANKA_1360300, respectively), ATP-dependent RNA helicase DDX6 (DOZI; PBANKA_1217700) and trailer hitch homolog (CITH; PBANKA_1301300) were identified as GBP2-interacting proteins (**Table 1**, **Supplementary Figure S3**). A phosphorylated adapter RNA export protein (PHAX) domain-containing protein (PBANKA_0506100), which is associated with mRNA export from the nucleus into the cytoplasm (Okamura et al., 2015), also interacted with GBP2 (**Table 1**), implying that an mRNA export pathway involving PHAX domain-containing proteins is present in malaria parasites (**Supplementary Figure S3**). However, no nuclear pore complex proteins or export receptor-like proteins were detected by IP-MS of GBP2::mCherry.

In the three independent comparative proteomics analyses of NAB2, 390, 410, and 536 proteins were detected. Among them, 30 proteins with at least three peptide spectral matches and a fold change ≥ 2.5 compared with the controls among three independent experiments were analyzed further (**Table 2**). Similar to GBP2::mCherry, the nuclear protein FoP domain-containing protein (PBANKA_1234500) was detected following IP-MS of NAB2::mCherry (**Table 2**). In addition, nuclear proteins such as ATP-dependent RNA helicase UAP56 (PBANKA_0306800) (Serpeloni et al., 2016) and polyadenylate-binding protein 2 (PBANKA_0824800) (Minns et al., 2018) interacted with NAB2 (**Table 2**). Notably, we found that transportin (PBANKA_1126400) interacted with NAB2 (**Table 2**), which implies that NAB2 shuttles between the nucleus and the cytoplasm (**Supplementary Figure S3**). No nuclear pore complex proteins or export receptor-like proteins were detected by IP-MS of NAB2::mCherry (**Table 2**).

### Identification of RNAs Directly Bound by GBP2 and NAB2

**Figure 4:**
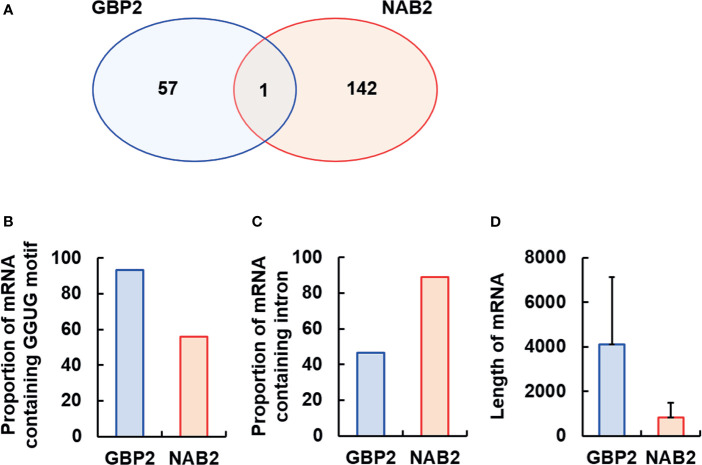
Analysis of the mRNAs bound by GBP2 or NAB2. To analyze the mRNAs bound by GBP2 or NAB2, mRNAs exhibiting < 500 transcripts per million in experimental subjects or > 500 transcripts per million in control subjects were excluded. **(A)** Venn diagram of mRNA sequences detected by RNA immunoprecipitation coupled to RNA sequencing of GBP2::mCherry and NAB2::mCherry parasites. **(B)** Percentage of mRNAs containing the GGUG motif among 58 and 143 mRNAs bound to GBP2 and NAB2, respectively. **(C)** Percentage of mRNAs containing an intron among 58 and 143 mRNAs bound to GBP2 and NAB2, respectively. **(D)** Length of 58 and 143 mRNAs bound to GBP2 and NAB2, respectively. Results are means ± standard deviation.

To identify RNAs bound by GBP2 and/or NAB2, we performed RIP-seq on mature schizonts- and gametocytes-lysates using anti-mCherry beads and sequenced the RNAs bound to GBP2 and NAB2 (**Figure 4**; **Supplementary Tables S2** and **S3**). As a control, RIP-seq using anti-GFP beads was also performed. In two independent RIP-seq assays of GBP2, 4,753 and 4,906 RNAs were detected. Among them, 58 mRNAs with < 500 transcripts per million (TPM) in both experimental subjects and > 500 TPM in controls (RNA immunoprecipitation in GBP2::mCherry parasites using anti-GFP beads coupled to RNA sequencing) were analyzed further (**Supplementary Table S2**). By contrast, 5,044 and 5,045 RNAs were detected in the two independent RIP-seq of NAB2, respectively. Among them, 143 mRNAs with < 500 TPM in both experimental subjects and > 500 TPM in controls (RNA immunoprecipitation in NAB2::mCherry parasites using anti-GFP beads coupled to RNA sequencing) were analyzed further (**Supplementary Table S3**).

Several mRNAs bound to GBP2 were expressed during the asexual development stage, such as copper-transporting ATPase (PBANKA_0416500) and a conserved *Plasmodium* protein of unknown function (PBANKA_0404000), or during the sexual stage, such as subtilisin-like protease 2 (PBANKA_0911700) and a conserved *Plasmodium* protein of unknown function (PBANKA_1029400) (**Supplementary Table S2**). Asexual stage mRNAs bound to NAB2 include translation initiation factor eIF-1A (PBANKA_0905600) and RNA lariat debranching enzyme (PBANKA_1354000); NAB2-bound sexual stage mRNAs include actin-related protein (PBANKA_0209300) and plasmepsin VII (PBANKA_0517600) (**Supplementary Table S3**). Moreover, we found that NAB2, but not GBP2, interacted with 40S and 60S ribosomal protein mRNAs, and that GBP2 and NAB2 typically bound to different mRNAs, with the exception of RNA-binding protein NOB1 (PBANKA_0720800) (**Figure 4A**; **Supplementary Figure S3**, **Tables S2** and **S3**).

The RRM2 domain of yeast GBP2 binds to RNAs containing the core motif GGUG and an intron (Hackmann et al., 2014; Martínez-Lumbreras et al., 2016). Among 58 mRNAs bound to GBP2, 54 (93.10%) contained the GGUG motif (**Figure 4B**). Among 143 mRNAs bound to NAB2, 80 (55.94%) contained the GGUG motif (**Figure 4B**). In contrast, the proportion of mRNAs containing an intron among the mRNAs that bound to NAB2 was 88.81%, higher than among mRNAs that bound to GBP2 (46.55%) (**Figure 4C**). The mRNAs bound to GBP2 were longer than those bound to NAB2 (**Figure 4D**). These results support our findings that GBP2 and NAB2 bound to different mRNAs in malaria parasites.

## Discussion

In this study, we investigated the roles of NAB2, THO4, NPL3, GBP2 and SR1, during the asexual and sexual developmental stages of *P. berghei* ANKA using reverse genetics. In yeast, deletion of NPL3, GBP2 or HRB1 (SR1 in *Plasmodium*) does not affect growth (Zander and Krebber, 2017). In malaria parasites, NPL3 and GBP2 were not essential for growth during the asexual stage. However, we found that SR1 plays an essential role in asexual development. Our results are consistent with those of a previous study, which revealed that SR1 plays an essential role in the asexual development of malaria parasites (Eshar et al., 2012). Furthermore, we found that GBP2 plays a more important role in male and female gametocyte development compared with THO4 and NPL3. These results confirm that GBP2 is involved in sexual development in malaria parasites.

We found that gametocyte development was less affected by *tho4* deletion compared with *gbp2* deletion. In *Drosophila*, always early (aly), a homolog of THO4, encodes a key protein in males both for the onset of spermatid differentiation and for the G2-meiosis I transition, but not for mRNA export (White-Cooper et al., 2000). Moreover, NPL3, but not GBP2 or HRB1, plays an essential role in meiotic gene expression in *S. cerevisiae* (Sandhu et al., 2021). Therefore, THO4 and NPL3 might be involved in mRNA export during meiosis, such as the mosquito stage of the malaria parasite.

In this study, we found that GBP2 was distributed throughout *P. berghei* ANKA cells. In yeast, GBP2 localizes to the nucleus, but not to the cytoplasm. Cytoplasmic mislocalization of yeast GBP2 was observed previously in gbp2 mutants, in which the binding sites of SR-specific protein kinases were exchanged (Windgassen and Krebber, 2003). Here, IP-MS of GBP2::mCherry revealed that GBP2 interacted with cytoplasmic proteins such as ALBA4 and DOZI (Munoz et al., 2017) in addition to nuclear proteins. Moreover, the NLS prediction server (http://www.moseslab.csb.utoronto.ca/NLStradamus/) indicated several nuclear localization signals for GBP2 (PRRRR; RR; KKDFRRDNRK), implying that GBP2 localizes not only to the nucleus but also to the cytoplasm. These results imply that the localization of GBP2 differs from that of yeast GBP2.

The results of IP-MS of GBP2::mCherry imply that GBP2 interacts with ALBA4, DOZI and CITH. DOZI and CITH are required for zygote development but not for gametocytogenesis (Mair et al., 2006; Mair et al., 2010). Furthermore, DOZI and CITH may be involved in translational repression during gametocytogenesis (Mair et al., 2006; Mair et al., 2010). ALBA4 is involved in sporozoite development and interacts with DOZI and CITH (Munoz et al., 2017). The reduced male and female gametocyte production by *gbp2* deletion mutants in this study implies that during the life cycle of malaria parasites, GBP2 functions during an earlier developmental stage than ALBA4, DOZI and CITH.

Our RIP-seq results revealed that GBP2 bound to mRNAs encoding proteins that are essential for asexual development; however, the parasitemia course was comparable in mice infected with Δ*gbp2 versus* control parasites. These findings imply that a factor other than GBP2 is involved in the export of these mRNAs. Here, we found that a phosphorylated adapter RNA export protein (PHAX) domain-containing protein associated with mRNA export *via* the CRM1/exportin pathway (Okamura et al., 2015) interacted with GBP2, implying that the GBP2-binding mRNAs that are essential for asexual development may be exported *via* the CRM1/exportin pathway in Δ*gbp2*.

In yeast, GBP2 is an adaptor protein for the Mex67/Mtr2 mRNA export receptor complex (Hackmann et al., 2014; Heath et al., 2016; Zander and Krebber, 2017). However, orthologues of Mex67 and Mtr2 are absent from malaria parasite genomes (Serpeloni et al., 2011; Avila et al., 2018). In this study, no export receptor-like proteins were detected by IP-MS of GBP2::mCherry. Exportin-1, an export receptor for PHAX domain-containing proteins, was also not detected by IP-MS of GBP2::mCherry. This indicates that detecting export receptors by IP-MS may be difficult due to weak binding between export receptors and adaptor proteins in malaria parasites.

NAB2 and SR1 were found to be essential for the survival of malaria parasites. Similar to SR1 (Eshar et al., 2015), NAB2 was located mainly at the periphery of the nucleus. However, no SR1-binding mRNAs (Eshar et al., 2015) were detected by RIP-seq of NAB2. Moreover, no SR1 homologues were detected by IP-MS of NAB2. Four homologues of proteins encoded by SR1-binding mRNAs (Eshar et al., 2015) (PBANKA_0401700, PBANKA_0404000, PBANKA_1212700 and PBANKA_1365300) were detected by RIP-seq of GBP2, while no SR1 homologues were detected by IP-MS of GBP2. These findings imply that NAB2, SR1 and GBP2 are associated with the export and quality control of different mRNAs.

In yeast, NAB2 is an adaptor protein for the Mex67/Mtr2 complex, similar to yeast SR proteins such as NPL3, GBP2 and HRB1 (Kehrer et al., 2018). The N-terminal domain of NAB2 also interacts with Mlp1, which associates with the nuclear pore complex and is involved in mRNA quality control and export (Xie and Ren, 2019). However, no nuclear pore complex proteins or export receptor-like proteins were detected by IP-MS of NAB2::mCherry. Fluorescence live cell imaging revealed that NAB2 localized not only to the nucleus but also to the nuclear membrane. These findings imply that NAB2 interacts with nuclear pore complex proteins in malaria parasites.

Here, we found that the protein transportin interacts with NAB2. Transportin mediates import into the nucleus in eukaryotic cells and recognizes the NLS of NAB2 (Chook and Süel, 2011). The NLS of yeast NAB2 comprises an N-terminal hydrophobic motif and a C-terminal PY-NLS (Soniat et al., 2013). NAB2 also contains a PY-NLS, implying that transportin is involved in the nuclear import of NAB2. These findings imply that NAB2 is involved in nuclear mRNA export in malaria parasites.

Our findings imply that NAB2 and GBP2 are involved in nuclear mRNA export in malaria parasites. Moreover, NAB2 and GBP2 function at different life-cycle stages. However, the terminal step of nuclear mRNA export in malaria parasites remains unclear. Future investigations should aim to identify export receptor and nuclear pore complex proteins that interact with NAB2 and SR proteins such as NPL3, GBP2 and SR1.

## Data Availability Statement

The datasets presented in this study can be found in online repositories. The names of the repository/repositories and accession number(s) can be found below: http://proteomecentral.proteomexchange.org/cgi/GetDataset PXD027302 https://www.ebi.ac.uk/arrayexpress/E-MTAB-10775, E-MTAB-10773.

## Ethics Statement

The animal study was reviewed and approved by the Experimental Animal Ethics Committee of Kyorin University School of Medicine.

## Author Contributions

MN designed research. MN and TF performed research. MN, TF, JM, and FK analyzed data. and MN and FK wrote the paper. All authors contributed to the article and approved the submitted version.

## Funding

This work was supported by a Grant–in–Aid for Scientific Research (C) from JSPS to MN (No. 18K07093 and No. 21K06997).

## Conflict of Interest

The authors declare that the research was conducted in the absence of any commercial or financial relationships that could be construed as a potential conflict of interest.

## Publisher’s Note

All claims expressed in this article are solely those of the authors and do not necessarily represent those of their affiliated organizations, or those of the publisher, the editors and the reviewers. Any product that may be evaluated in this article, or claim that may be made by its manufacturer, is not guaranteed or endorsed by the publisher.

## Supplementary Material

Supplementary Table S1 Sequence of primers used in this study.

Supplementary Table S2 RNA immunoprecipitation coupled to RNA-sequencing of GBP2::mCherry parasites. Protein-mRNA complex was extracted from NAB2::mCherry schizont- and gametocyte-enriched cultures after incubation for 22 h. mRNAs with < 500 transcripts per million (TPM) in experimental subjects and > 500 TPM in controls (RNA immunoprecipitation in GBP2::mCherry parasites using anti-GFP beads coupled to RNA sequencing) were excluded. Experiments were performed in duplicate; similar results were obtained from each experiment. Representative data are shown. PlasmoGEM indicates the phenotype of parasite multiplication during the blood stage of *P. berghei* ANKA (http://plasmogem.sanger.ac.uk/phenotypes). Expression profile indicates the developmental stage at which expression of mRNA was mainly observed during the blood stage of *P. berghei* ANKA (https://plasmodb.org/plasmo/app).

Supplementary Table S3 RNA immunoprecipitation coupled to RNA-sequencing of NAB2::mCherry parasites. Protein-mRNA complexes were extracted from NAB2::mCherry schizont- and gametocyte-enriched cultures after incubation for 22 h. mRNAs with < 500 transcripts per million (TPM) in experimental subjects and > 500 TPM in controls (RNA immunoprecipitation in NAB2::mCherry parasites using anti-GFP beads coupled to RNA sequencing) were excluded. Experiments were performed in duplicate; similar results were obtained from each experiment. Representative data are shown. PlasmoGEM indicates the phenotype of parasite multiplication during the blood stage of *P. berghei* ANKA (http://plasmogem.sanger.ac.uk/phenotypes). Expression profile indicates the developmental stage at which expression of mRNA was mainly observed during the blood stage of *P. berghei* ANKA (https://plasmodb.org/plasmo/app).

Supplementary Figure S1 Schematic representation of the gene-targeting vector used to disrupt NPL3, THO4, NAB2 and SR1. Gene disruption was performed by double-crossover homologous recombination. The gene disruption vectors contained human dihydrofolate reductase–thymidylate synthase (*hdhfr*) and the 5′ and 3′ flanking regions of target genes. hDHFR expression was controlled by the elongation factor-1 promoter (PBANKA_113340). Arrows denote primers specific for the 5′ and 3′ regions of target genes. **(A)** Introduction of the hDHFR-expressing cassette into the *nab2* locus of wild-type (WT) *P. berghei* ANKA. **(B)** Introduction of the hDHFR-expressing cassette into the *tho4* locus of WT *P. berghei* ANKA. *tho4*-specific primer sets were used. PCR products were digested with BamHI (red arrowhead) to distinguish between wild-type *P. berghei* ANKA (1720 and 1090 bp fragments) and *tho4* deletion mutants (Δ*tho4*) (2202 and 508 bp fragments). **(C)** Introduction of the hDHFR-expressing cassette into the *npl3* locus of WT *P. berghei* ANKA. *npl3*-specific primer sets were used. PCR products were digested with BamHI (red arrowhead) to distinguish between WT (3088 bp) and *npl3* deletion mutants (Δ*npl3*) (2337 and 826 bp fragments). **(D)** Introduction of the hDHFR-expressing cassette into the *sr1* locus of WT *P. berghei* ANKA. To generate deletion mutants, two independent transfections were performed in each line. Note: *nab2* and *sr1* deletion mutants could not be generated.

Supplementary Figure S2 Generation of parasites to investigate localizations of GBP2 and NAB2. Schematic representation of the gene-targeting vectors used to express GBP2::mCherry, NAB2::mCherry and NUP205::GFP fusion proteins. The gene-targeting vector contained *mCherry* or green fluorescent protein (*gfp*), human dihydrofolate reductase–thymidylate synthase (*hdhfr*) or mutated *hdhfr* (*hdhfs*), and the 3′ regions of the target genes. The *gbp2::mCherry*, *nab2::mCherry* and *nup205::gfp* genes were under the control of native promoters. *hdhfr* and *hdhfs* were under the control of the elongation factor-1 (PBANKA_113340) promoter. Arrows denote primers specific for the 5′ and 3′ regions of gene-targeting vectors. **(A)** Introduction of the *gbp2::mCherry* cassette into the 3′ region of the *gbp2* locus of wild-type (WT) *P. berghei* ANKA. The correct integration of *mCherry* and *hdhfp* into the 3′ region of *gbp2* was confirmed by PCR (WT fragment, 2389 bp; GBP2::mCherry fragment, 2017 bp). **(B)** Introduction of the *nab2::mCherry* cassette into the 3′ region of the *nab2* locus of WT *P. berghei* ANKA. LD, limiting dilution. Pre-LD indicates parasites transfected before LD. Correct integration of *mCherry* and *hdhfp* into the 3′ regions of *nab2* was confirmed by PCR (WT fragment, 1744 bp; NAB2::mCherry fragment, 4544 bp). **(C)** Introduction of the *nup205::gfp* cassette into the 3′ region of the *nup205* locus of NAB2::mCherry *P. berghei* ANKA. The correct integration of *gfp* and *hdhps* into the 3′ region of *nup205* was confirmed by PCR (WT fragment, 1624 bp; NUP205::GFP fragment, 6153 bp).

Supplementary Figure S3 Schematic representation of mRNA export by GBP2 and NAB2 in malaria parasites. **(A)** GBP2 and NAB2 typically bound to different mRNAs in malaria parasites. **(B)** No nuclear pore complex proteins or export receptor-like proteins were identified in this study. **(C)** The GBP2-binding mRNAs may also be exported *via* the CRM1/exportin pathway. **(D)** GBP2 interacts with ALBA4, DOZI and CITH. **(E)** Transportin is involved in the nuclear import of NAB2.

## Abbreviations

DOZI, ATP-dependent RNA helicase DDX6; CRM1, Chromosome maintenance region 1; ALBA, DNA/RNA-binding protein Alba; GBP2, G-strand binding protein 2; HRB1, Hypothetical RNA-binding protein; IP-MS, IP coupled to MS; IP, Immunoprecipitation; MS, Mass spectrometry; NLSs, Nuclear localization signals; NAB2, Nuclear poly (A) binding protein 2; NPCs, Nuclear pore complexes; NPL3, Nucleolar protein 3; Nups, Nucleoporins; FG-Nups, Nups with frequent phenylalanine-glycine repeats; RIP-seq, RIP coupled to RNA-sequencing; RIP, RNA immunoprecipitation; RRMs, RNA recognition motifs; RBPs, RNA-binding proteins; SR, Ser/Arg-rich; THO4, THO complex subunit 4; CITH, Trailer hitch homolog; TPM, Transcripts per million; YRA1, Yeast RNA Annealing protein.
