# Supplementary figures and images for: Roles and Cellular Localization of GBP2 and NAB2 During the Blood Stage of Malaria Parasites
Source: Front Cell Infect Microbiol. 2021 Sep 15;11:737457. doi: 10.3389/fcimb.2021.737457 (PMC8479154; doi:10.3389/fcimb.2021.737457)

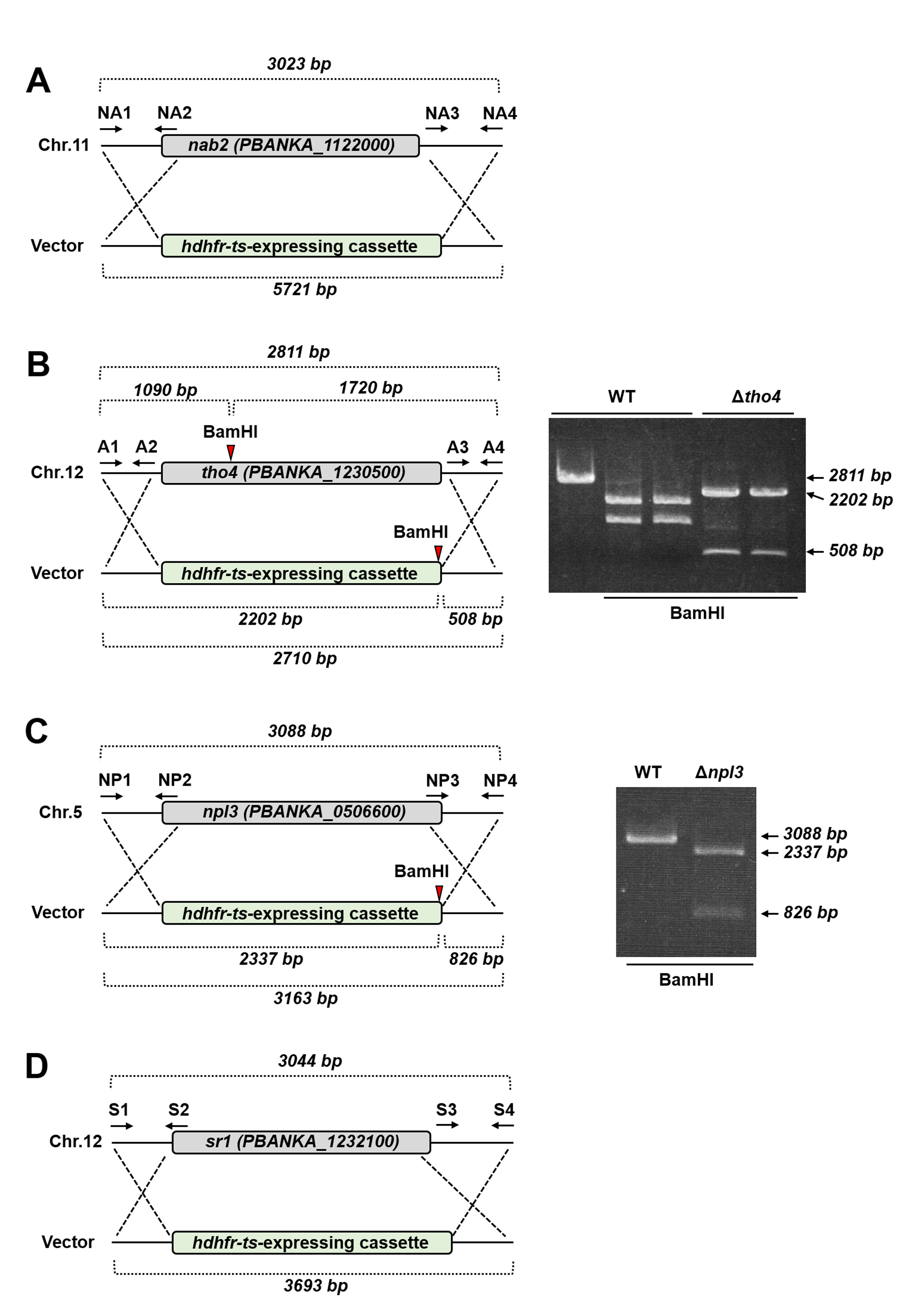

Supplement: Supplementary Figure S1 — Schematic representation of the gene-targeting vector used to disrupt NPL3, THO4, NAB2 and SR1. Gene disruption was performed by double-crossover homologous recombination. The gene disruption vectors contained human dihydrofolate reductase–thymidylate synthase (hdhfr) and the 5′ and 3′ flanking regions of target genes. hDHFR expression was controlled by the elongation factor-1 promoter (PBANKA_113340). Arrows denote primers specific for the 5′ and 3′ regions of target genes. (A) Introduction of the hDHFR-expressing cassette into the nab2 locus of wild-type (WT) P. berghei ANKA. (B) Introduction of the hDHFR-expressing cassette into the tho4 locus of WT P. berghei ANKA. tho4-specific primer sets were used. PCR products were digested with BamHI (red arrowhead) to distinguish between wild-type P. berghei ANKA (1720 and 1090 bp fragments) and tho4 deletion mutants (Δtho4) (2202 and 508 bp fragments). (C) Introduction of the hDHFR-expressing cassette into the npl3 locus of WT P. berghei ANKA. npl3-specific primer sets were used. PCR products were digested with BamHI (red arrowhead) to distinguish between WT (3088 bp) and npl3 deletion mutants (Δnpl3) (2337 and 826 bp fragments). (D) Introduction of the hDHFR-expressing cassette into the sr1 locus of WT P. berghei ANKA. To generate deletion mutants, two independent transfections were performed in each line. Note: nab2 and sr1 deletion mutants could not be generated. [file Image_1.tif]

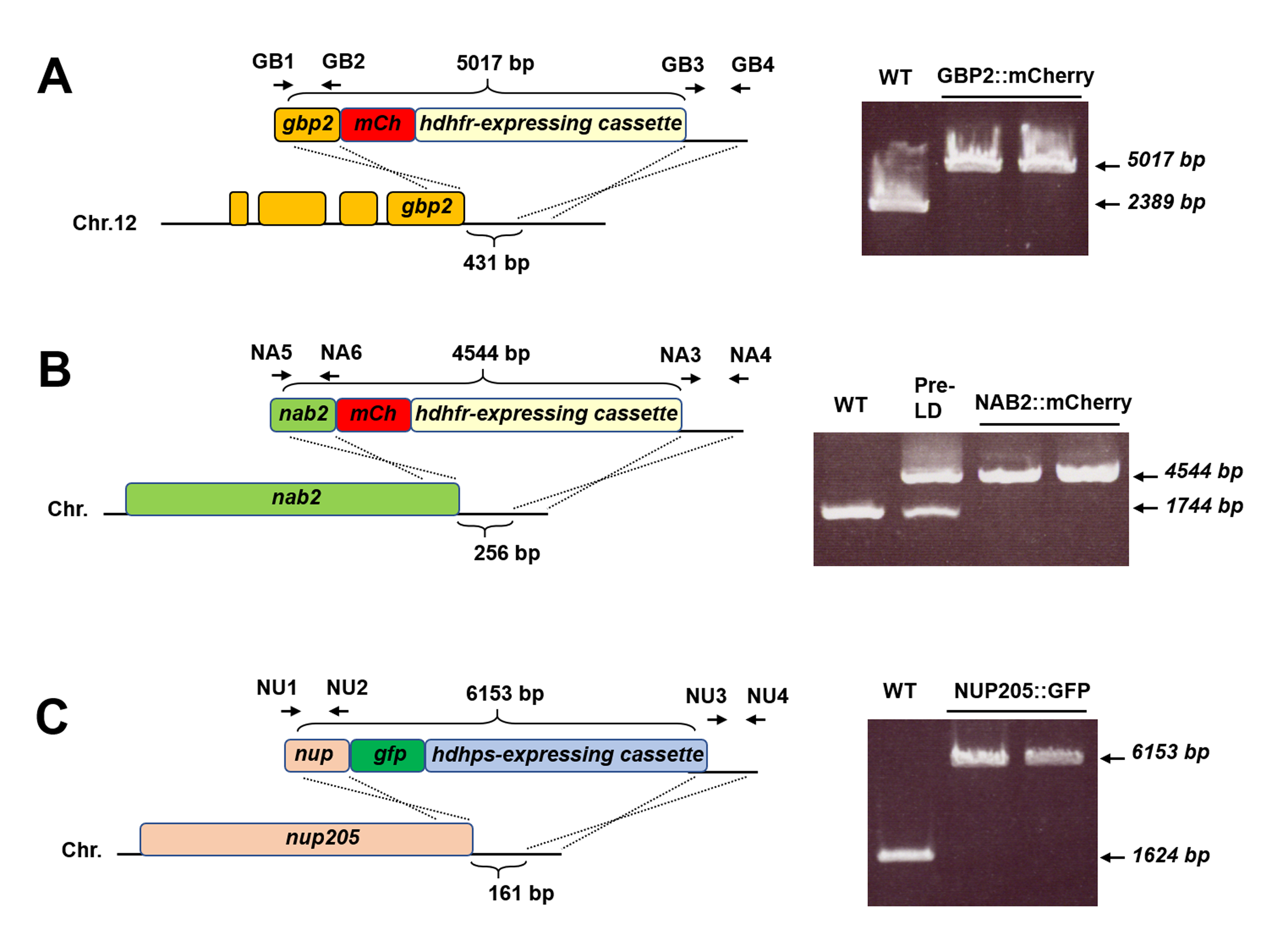

Supplement: Supplementary Figure S2 — Generation of parasites to investigate localizations of GBP2 and NAB2. Schematic representation of the gene-targeting vectors used to express GBP2::mCherry, NAB2::mCherry and NUP205::GFP fusion proteins. The gene-targeting vector contained mCherry or green fluorescent protein (gfp), human dihydrofolate reductase–thymidylate synthase (hdhfr) or mutated hdhfr (hdhfs), and the 3′ regions of the target genes. The gbp2::mCherry, nab2::mCherry and nup205::gfp genes were under the control of native promoters. hdhfr and hdhfs were under the control of the elongation factor-1 (PBANKA_113340) promoter. Arrows denote primers specific for the 5′ and 3′ regions of gene-targeting vectors. (A) Introduction of the gbp2::mCherry cassette into the 3′ region of the gbp2 locus of wild-type (WT) P. berghei ANKA. The correct integration of mCherry and hdhfp into the 3′ region of gbp2 was confirmed by PCR (WT fragment, 2389 bp; GBP2::mCherry fragment, 2017 bp). (B) Introduction of the nab2::mCherry cassette into the 3′ region of the nab2 locus of WT P. berghei ANKA. LD, limiting dilution. Pre-LD indicates parasites transfected before LD. Correct integration of mCherry and hdhfp into the 3′ regions of nab2 was confirmed by PCR (WT fragment, 1744 bp; NAB2::mCherry fragment, 4544 bp). (C) Introduction of the nup205::gfp cassette into the 3′ region of the nup205 locus of NAB2::mCherry P. berghei ANKA. The correct integration of gfp and hdhps into the 3′ region of nup205 was confirmed by PCR (WT fragment, 1624 bp; NUP205::GFP fragment, 6153 bp). [file Image_2.tif]

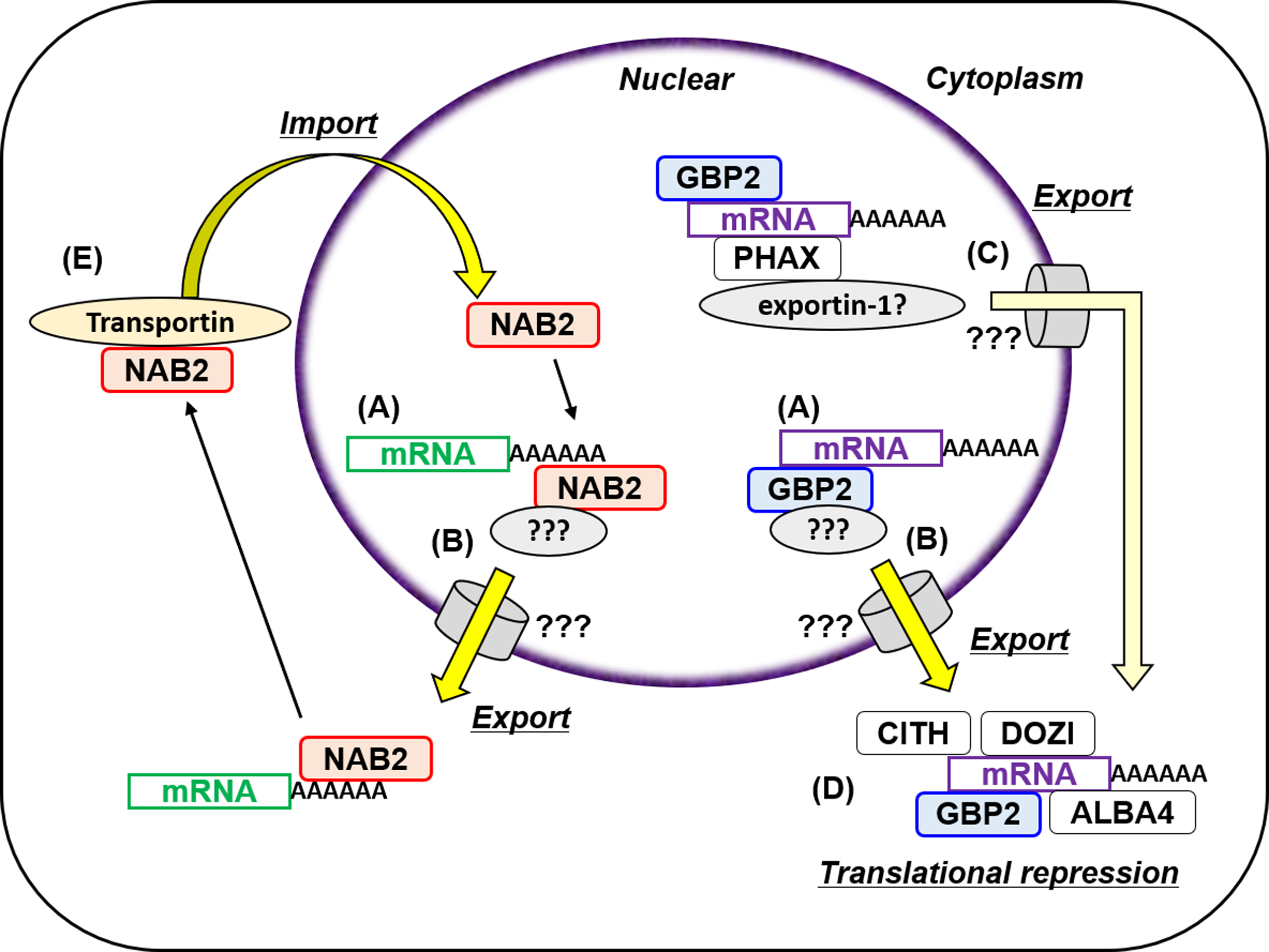

Supplement: Supplementary Figure S3 — Schematic representation of mRNA export by GBP2 and NAB2 in malaria parasites. (A) GBP2 and NAB2 typically bound to different mRNAs in malaria parasites. (B) No nuclear pore complex proteins or export receptor-like proteins were identified in this study. (C) The GBP2-binding mRNAs may also be exported via the CRM1/exportin pathway. (D) GBP2 interacts with ALBA4, DOZI and CITH. (E) Transportin is involved in the nuclear import of NAB2. [file Image_3.tif]
